# Spatial tumour characteristics as an indirect marker of metabolic dysregulation: evaluation for non-invasive IDH-genotyping of glioma using hybrid [18 F]FET-PET/MRI

**DOI:** 10.1007/s00259-025-07520-8

**Published:** 2025-08-28

**Authors:** Johannes Lohmeier, Jenny Meinhardt, Helena Radbruch, Mauricio Reyes, Winfried Brenner, Anna Tietze, Marcus R. Makowski

**Affiliations:** 1 Institute of Neuroradiology, Campus Charité Mitte (CCM), Charité - Universitätsmedizin Berlin, Freie Universität Berlin and Humboldt Universität zu Berlin, Charitéplatz 1, 10117 Berlin, Germany; 2 Department of Neuropathology, Campus Charité Mitte (CCM, Charité - Universitätsmedizin Berlin, Freie Universität Berlin and Humboldt Universität zu Berlin, Charitéplatz 1, Berlin, 10117 Germany; 3 ARTORG Center for Biomedical Engineering Research, Medical Image Analysis Group, University of Bern, Murtenstrasse 50, Bern, CH-3008 Switzerland; 4 Department of Nuclear Medicine, Charité - Universitätsmedizin Berlin, Freie Universität Berlin and Humboldt Universität zu Berlin, Augustenburger Platz 1, Berlin, 13353 Germany; 5 Department of Radiology, Technical University Munich, Ismaninger Str. 22, München, 81675 Germany; 6 Department of Radiation Oncology, University Hospital of Bern, Bern, Switzerland

**Keywords:** Neuro-oncology, PET/MRI, Predictive IDH-genotyping, Radiogenomic biomarker, Spatial phenotyping, Spatial tumour characteristics

## Abstract

**Purpose:**

The isocitrate dehydrogenase (IDH) genotype is crucial for diagnosing and managing adult-type diffuse glioma. We investigated spatial tumour characteristics in treatment-naïve glioma using an U-Net-based CNN and evaluated associations with metabolic dysfunction and IDH genotype.

**Methods:**

Between 2015 and 2024 patients with confirmed contrast-enhancing glioma were pre-operatively investigated using MRI or [18 F]FET PET/MRI. Automated morphometry using a U-Net-based CNN on standard MRI sequences (T1c, T1, T2, FLAIR) was performed. Contrast-enhancing tumour fraction (CTF), metabolic tumour volume (MTV), total tumour volume (TTV) were determined. Dice coefficient assessed volume intersections. Comparative and statistical analyses included non-parametric tests, ROC curves, regression, and correlation.

**Results:**

A total of 180 patients (male, 114; female, 66; age, M ± SD = 54 ± 15y; IDH-mutant, 63; IDH wild-type, 117) with treatment-naïve glioma were evaluated. [18 F]FET-PET metabolic activity correlated significantly with CTF (*p* < .05). IDH-mutant gliomas had lower CTF (*p* < .001) due to higher non-enhancing tumour mass (*p* < .001) relative to the enhancing mass, unlike IDH wild-type glioblastoma. The CTF predicted IDH genotype with high accuracy (AUC = 0.85, sensitivity 78%, specificity 90%) across datasets. Combining CTF with patient age or SUVmax further improved the classification (ΔAUC = 0.12, *p* = .02; ΔAUC = 0.09, *p* > .05). Subgroup analyses showed consistent performance across IDH-mutant subtypes. MTV from [18 F]FET-PET exceeded structurally apparent TTV (*p* = .033).

**Conclusion:**

Spatial mapping of treatment-naïve glioma identified a non-invasive biomarker, which is linked to metabolic dysfunction and enabled robust IDH-genotype classification from standard MRI, suggesting a central role for radiogenomic assessment in adult-type diffuse gliomas prior to surgery.

**Supplementary Information:**

The online version contains supplementary material available at 10.1007/s00259-025-07520-8.

## Introduction

**Fig. 1:**
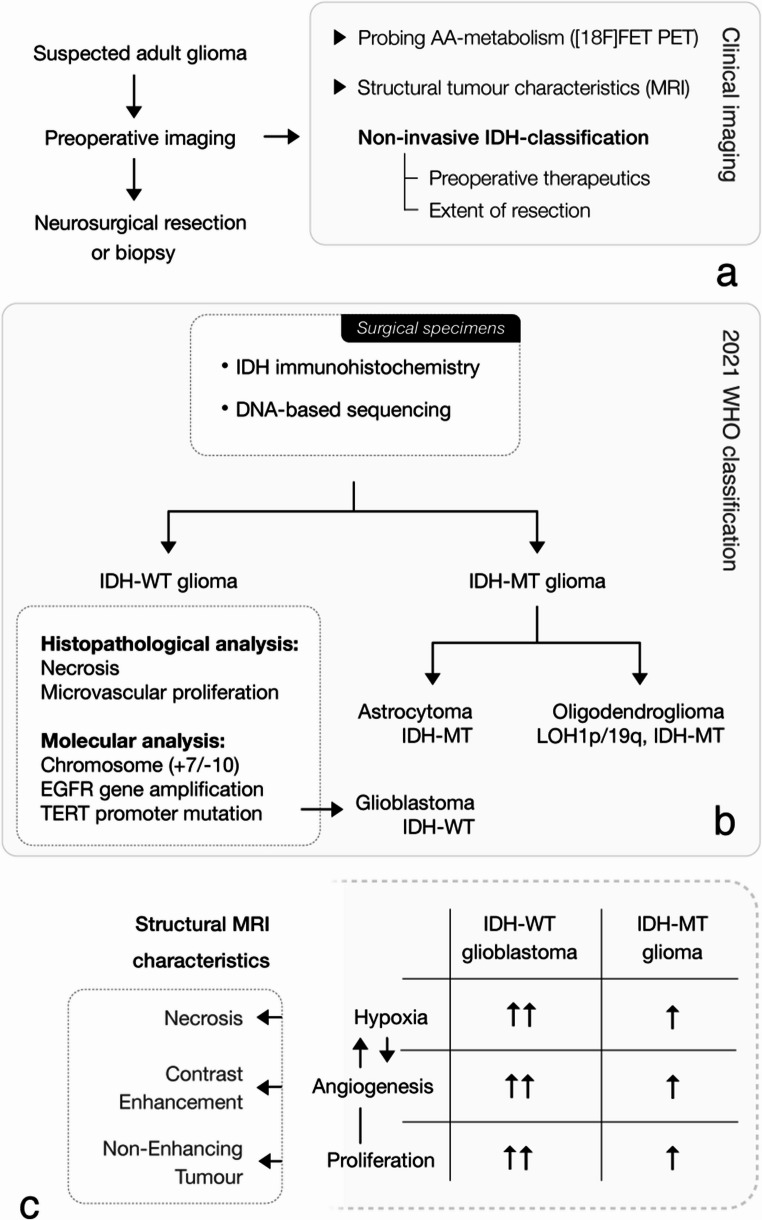
Clinical role of IDH-genotyping in adult glioma.** A** In adult gliomas, IDH-genotype differentiates the group of IDH-mutated astrocytoma and oligodendroglioma from IDH wild-type glioblastoma. Molecular stratification prior to surgical resection opens a window-of-opportunity for neo-adjuvant treatment using molecular targeting strategies and may guide the extent of resection. **B** Metabolic dysfunction in glioma drives macrostructural changes visible on imaging. Severe hypoxia results from rapid tumour growth and insufficient vascular supply, promoting aberrant angiogenesis and leaky vessels, which may become apparent as contrast enhancement on MRI. Slow-growing, IDH-mutant gliomas typically show larger non-enhancing regions, while aggressive, IDH wild-type glioblastomas exhibit extensive hypoxia, necrosis, and predominantly contrast-enhancing tumour mass. IDH-MT/-WT = Isocitrate dehydrogenase mutated/wild-type. EOR = Extent of resection. EGFR = Epidermal Growth Factor Receptor. TERT = telomerase reverse transcriptase promoter mutation

Adult-type diffuse gliomas account for the majority of malignant primary brain tumours, marking a principal cause of death in Central Nervous System (CNS) cancers [1]. Recent updates to the WHO Classification of CNS Tumours emphasised the critical role of genetic characteristics [2]. Notably, mutations of the isocitrate dehydrogenase (IDH) genes delineate a subset of adult-type glioma with a more favourable prognostic outlook compared to the aggressive IDH wild-type glioblastoma (see Fig. 1) – making the IDH genotype a central determinant for the diagnosis, therapy and management of patients with CNS cancer. IDH-mutated gliomas characterised by an additional chromosomal translocation involving an imbalance between chromosomes 1 and 19 are classified as oligodendroglioma, while the absence of a chromosomal 1p/19q co-deletion distinguish astrocytoma. IDH mutations manifest at the onset of gliomagenesis and act as a critical driving force of cancer progression [3] through disruption of DNA and histone methylation, DNA repair, and cellular differentiation by reprogramming of the citric acid cycle [4].

To date, the multimodal standard of care in IDH-mutant and wild-type gliomas comprises surgical resection and radio-chemotherapy. However, several targeted therapeutics emerged in the last decade, which open up a promising avenue for personalised treatment of patients with IDH-mutant gliomas, including selective inhibitors of mutant IDH, an IDH1-R132H-specific vaccine, immunotherapeutics (targeting the quiescent immune-environment), inhibitors of glutaminase and poly-ADP ribose polymerase or demethylating agents [1, 5, 6]. Recently, the FDA approved vorasidenib, an oral dual IDH1/IDH2-inhibitor, for patients with grade 2 gliomas harbouring IDH1 or IDH2 mutations, based on the promising results of the INDIGO trial [7]. The oral IDH1-inhibitor olutasidenib (FT-2102) is another promising therapeutic agent, which demonstrated effective disease control in enhancing, high-grade and low-grade (relapsed/refractory) gliomas in a phase Ib/II trial [8]. With the rising availability of targeted therapeutics, there is a growing and clinically unmet need for non-invasive techniques that enable IDH-prediction prior to surgical intervention (see Fig. 1) – thereby, preparing the ground for targeted neo-adjuvant treatment strategies and the guidance of resection margin. In this context, patients with IDH-mutant glioma were shown to benefit from supra-maximal resection, with multi-year gains in survival [9–11], whereas in IDH wild-type glioblastoma, the same approach yields only modest survival gains [12]. Given the glioblastoma’s aggressive nature [1], the risk–benefit profile is less favourable, as potential neurological deficits may outweigh limited clinical benefit. Nonetheless, reliable techniques for non-invasive IDH-classification remain scarce and are rarely implemented in clinical practice [13] – thus, the molecular profile is typically unknown prior to neurosurgical resection or biopsy.

Due to their different molecular underpinnings, IDH wild-type glioblastoma and IDH-mutant glioma exhibit distinct patterns of cellular, structural, immune, and, more importantly, metabolic phenotypes [3, 4, 14]. Metabolic imaging using radio-labelled amino acids was shown to be useful for predicting IDH-genotype [15–18] by utilising tumour metabolic dysfunction. Metabolic reprogramming drives malignant features such as aberrant vascularisation and tissue necrosis, which manifest as contrast enhancement and necrotic regions on imaging (see Fig. 1). Since IDH mutational status is a key regulator of tumour metabolism, we hypothesised that the contrast-enhancing tumour fraction (CTF) would reflect metabolic dysfunction and predict IDH mutational status. Therefore, we performed automated MRI morphometry of treatment-naïve glioma using an U-Net-based convolutional neural network (CNN) in a model building dataset to determine the CTF as a non-invasive imaging biomarker. Then, we investigated our hypothesis using O-(2-[18 F]fluoroethyl)-L-tyrosine (FET) PET/MRI and evaluated an association with metabolic activity and IDH prediction in a patient cohort with treatment-naïve glioma.

## Patients and methods

### Study design and patients

**Fig. 2:**
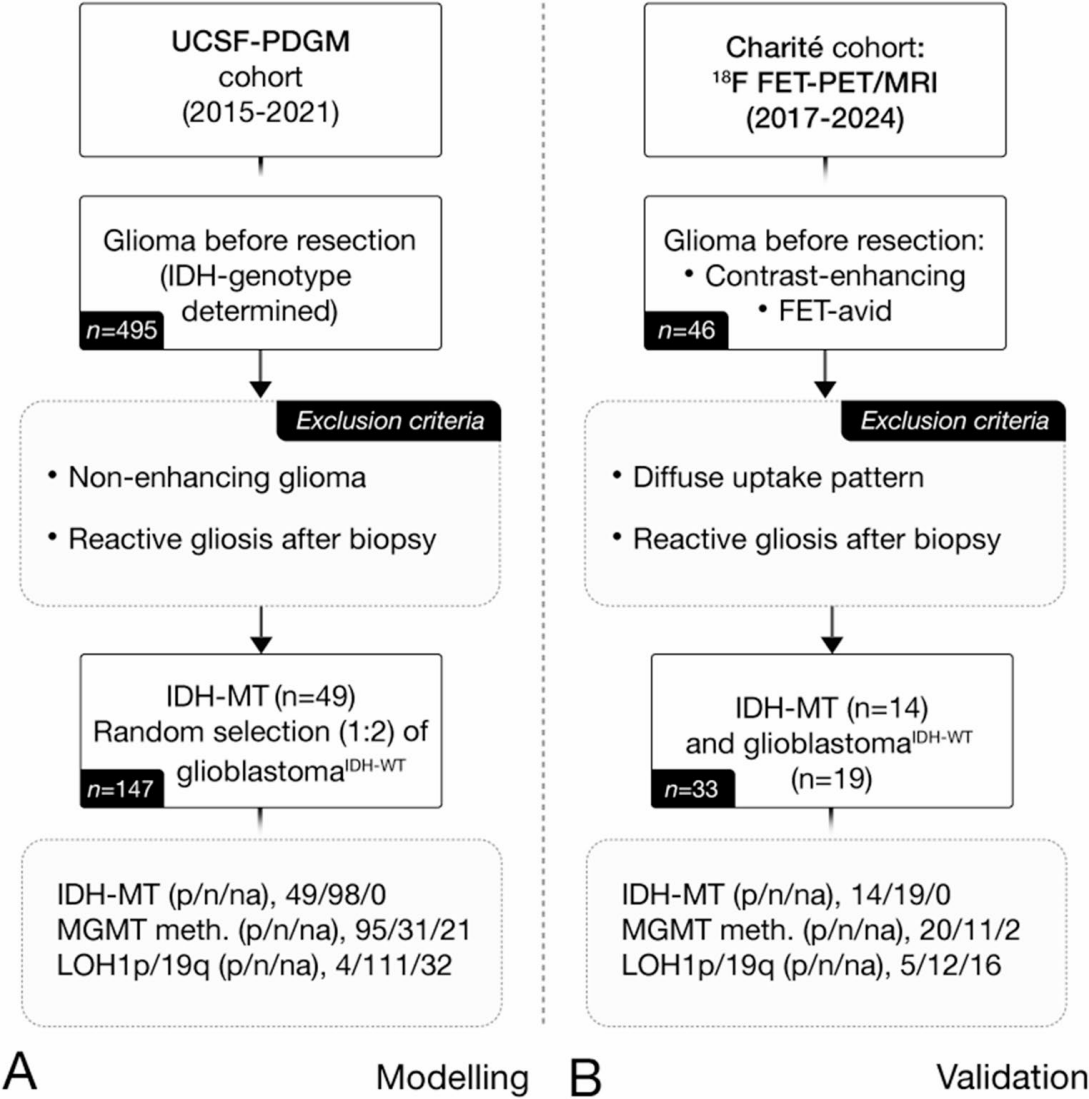
Patient selection flowchart. Selection of participants for the model building **A** and validation cohort **B**. Patients with open biopsy, partial resection or reactive gliosis after biopsy were excluded. IDH-MT/-WT = Isocitrate dehydrogenase mutated/wild-type, MGMT = O6-methylguanine-DNA-methyltransferase, LOH1p/19q = Loss of heterozygosity of 1p/19q. p/n/na = positive/negative/not applicable

This retrospective clinical cohort study was conducted according to the principles of the Helsinki Declaration and approval from the institutional ethics board (EA2/019/23) was obtained. For validation, patients who received hybrid [18 F]FET-PET/MRI between 2017 and 2024 at the Charité University Hospital Berlin, Germany for suspected contrast-enhancing brain tumour, subsequently diagnosed with a glioma following the 2021 WHO classification [2], were investigated. For model building, the University of California San Francisco Preoperative Diffuse Glioma MRI (UCSF-PDGM) cohort [19, 20] was used. The UCSF-PDGM cohort comprises 495 patients with histopathologically confirmed CNS WHO grade 2–4 diffuse adult-type gliomas following the 2021 WHO Classification of CNS Tumours [2] who received standardised MR-imaging prior to neurosurgical resection between 2015 and 2021. Considering that glioblastoma is by far the most common adult glioma, with astrocytomas and oligodendrogliomas occurring much less frequently, we randomly selected glioblastoma in a ratio of 2:1, reflecting the natural prevalence [21]. In addition, the recently updated TCGA-LGG cohort [22] provided additional patients with contrast-enhancing astrocytoma and oligodendroglioma for comparative analysis of CTF, excluding patients with “mixed glioma” and those with non-standard MRI protocols (missing T1, T1c, T2, or FLAIR sequences), as shown in Supplemental Figs. 1 and 2. Patients with open biopsy, partial resection or reactive gliosis after biopsy were excluded to mitigate the risk of confounding uptake or signal changes that could affect segmentation and quantitative measurements. See Fig. 2; Table 1 for further details.

**Table 1:** Characteristics of the patient cohorts

	UCSF-PDGM	Charité
Participants (n)	147	33
Age (M ± SD in y)	54 ± 15	54 ± 16
Sex (male/female)	96/51	18/15
IDH-mutational status(positive/negative/NA)	49/98/0	14/19/0
MGMT promoter methylation status(positive/negative/NA)	95/31/21	20/11/2
LOH1p/19q (positive/negative/NA)	4/111/32	5/12/16
CNS WHO grade	125 CNS WHO grade 4,	24 CNS WHO grade 4,
	16 CNS WHO grade 3,	5 CNS WHO grade 3,
	6 CNS WHO grade 2	4 CNS WHO grade 2

*IDH* Isocitrate Dehydrogenase, *MGMT* O6-methylguanine-DNA-methyltransferase, *LOH1p/19q* Loss of heterozygosity of 1p/19q, *CNS* Central Nervous System, *WHO* World Health Organization, *NA* not applicable

## Neuropathological analysis

For the UCSF-PDGM cohort, neuropathological workup included the identification of IDH mutations (IDH-MT/WT, mutant/wild-type) using either conventional Sanger sequencing or next-generation genetic sequencing, testing for 1p/19q co-deletion (LOH1p/19q+/-) via fluorescence in situ hybridisation and O6-methylguanine-DNA-methyltransferase (MGMT+/-) promoter methylation status (for all grade 3 and 4 tumours) assessed using an in-house methylation polymerase chain reaction assay [19]. For the Charité cohort, detection of IDH mutations was performed by immunostaining and/or pyrosequencing, 1p/19q co-deletion using EPIC DNA methylation arrays and MGMT promoter methylation using EPIC DNA methylation arrays or via pyrosequencing analysed using the PyroMark Q24 (Qiagen) and the Pyromark MGMT kit from FFPE tissue specimens during routine diagnostic workup. To mitigate the impact of the different molecular methods, we ensured that all classifications were based on validated, clinically standard techniques accepted by the WHO [2].

### PET and MRI acquisition

The MRI acquisition protocols of the UCSF-PDGM included a T2-weighted, T2-weighted fluid attenuated inversion recovery (FLAIR) and a pre- and post-contrast T1-weighted sequence upon administration of gadolinium-based contrast agent (GBCA) according to the patient’s total body-weight on a clinical 3T-MRI scanner [19]. In the Charité cohort, patients underwent simultaneous PET and 3 T MRI acquisitions using a Siemens MAGNETOM Biograph mMR system (Siemens Healthcare, Erlangen, Germany) [23] in list-mode for up to 60 min following intravenous administration of [18 F]FET (163 ± 23 MBq; 180 MBq standard dose or weight-adjusted dose for < 60 kg) and GBCA (Gadovist^®^, Bayer Pharma AG, Berlin, Germany) administered at 0.1 mmol/kg based on total body weight. A minimum 4-hour fast was recommended prior to PET. The PET/MRI acquisition protocol included a transversal T1-weighted ultrashort echo time (UTE) for attenuation and scatter correction, a native T2-weighted, a native T2-FLAIR-weighted, a pre-contrast T1-weighted and a post-contrast T1-weighted magnetization-prepared rapid gradient echo (MPRAGE) sequence. PET acquisition was reconstructed into transaxial slices using an iterative ordered-subset expectation maximization algorithm (OSEM, 3 iterations and 21 subsets; matrix-size = 344 × 344 × 127; voxel-size = 1.0 × 1.0 × 2.3 mm3; gaussian-filter = 3 mm). Emission data was corrected for decay, randoms, dead time, scatter and attenuation.

### PET and MRI analysis

Automated MRI morphometry was performed using an U-Net-based convolutional neural network (CNN) [24]. DeepBraTumIA is a (publicly available) research tool for automated brain tumour segmentation, which employs a 3D U-Net-based CNN to delineate tumour compartments from standard MRI sequences (T1, T1c, T2, FLAIR). It preprocesses images through intensity normalisation, skull-stripping (HD-BET [25]), and rigid registration (SimpleITK [26]), followed by voxel-wise classification to segment contrast-enhancing tumour (CE), edema, and a combined necrotic/non-enhancing region (non-CE). The non-enhancing tumour component in pre-operative glioma imaging was merged with the necrotic tissue label, aligning with BRATS (Brain Tumor Segmentation Challenge) recommendations to mitigate inter-rater variability. While this method enhances the robustness of volumetric analysis, it compromises the specificity in defining the non-enhancing tumour mass. NS-HGlio, which is the commercial successor to DeepBraTumIA, was previously validated, as described in the study by Abayazeed et al. [27].

The contrast-enhancing (CE) and combined necrotic and non-enhancing (non-CE) compartments were visually inspected for plausibility and, where applicable, automated segmentations were slightly adapted, such as the exclusion of major (non-leaky) vasculature, imaging artefacts, choroid plexus, small stereotactic biopsy defects, areas of haemorrhage, and minor adaptations to include more subtle contrast enhancement or non-enhancing tumour. The CTF was defined as the mass of CE relative to the TTV (Eq. 1). The total tumour volume (TTV) is the sum of the non-CE and CE mass. Based on our hypothesis that the internal composition of the solid tumour may be associated with metabolic reprogramming and the IDH-genotype, peritumoral edema was excluded from analysis.

Quantitative analysis of hybrid PET/MRI was performed using OsiriX MD 14.01 (Pixmeo SARL, Bernex Switzerland). [18 F]FET uptake measurement was performed in an automated manner using iso-contouring based on attenuation-corrected [18 F]FET tracer uptake. For the definition of metabolic tumour volume (MTV), an absolute threshold method with 1.8 times the mean uptake of the healthy contralateral hemisphere (spherical VOI including white and gray matter) was used according to the adapted guideline recommendation [28]. Where applicable, volume-of-interests (VOIs) were corrected for the exclusion of non-tumoral uptake. VOIs from hybrid [18 F]FET-PET/MRI were transformed to atlas space for further analysis.

1 $$CTF=\frac{CE}{TTV}$$

### Statistics

Statistical analysis was performed using Prism v10 (GraphPad Software, San Diego, CA, USA) and MedCalc v23.0.1 (MedCalc Software Ltd, Ostend, Belgium). Mann-Whitney-U tests (with Holm-Šídák multiple-comparison testing where applicable) or Kruskal–Wallis test were used for comparisons of independent groups. Wilcoxon signed-rank testing was used for matched pairs. Receiver-Operating-Characteristic (ROC) analysis was performed (using DeLong method) reporting area under the curve (AUC), 95%-CIs, and *p*-value. Sensitivity and specificity were reported for the best cutoff point independent of the prevalence determined using the Youden index. Logistic regression was used to model binary outcome. Measurements were correlated and evaluated using the non-parametric Spearman correlation coefficient. Intersections between volumes were computed based on Dice coefficient (Eq. 2). A Dice coefficient of 0 indicates no overlap; an index of 1 implies perfect agreement among volumes. In all tests, a *p*-value < .05 was considered statistically significant.

2 $$DSC=2\ast\frac{\left|X\cap Y\right|}{\left|X\right|+\left|Y\right|}$$

## Results

### Patient population

A total of 180 patients (male, 114; female, 66; age, M ± SD = 54 ± 15y; IDH-mutant, 63; IDH wild-type, 117) with treatment-naïve glioma were evaluated. Within the UCSF-PDGM cohort, 147 treatment-naïve glioma patients (male, 96; female, 51; M ± SD = 54 ± 15y) were studied. This included 49 patients with contrast-enhancing IDH-mutated gliomas – 27 CNS WHO grade 4, 16 CNS WHO grade 3, 6 CNS WHO grade 2 – of which 8% were oligodendrogliomas and 92% were astrocytomas, alongside 98 randomly chosen IDH wild-type glioblastoma cases (CNS WHO grade 4) to reflect their prevalence (as discussed above). The Charité cohort comprised 33 patients (male, 18; female, 15; age, M ± SD = 54 ± 16 y) with [18 F]FET-avid and contrast-enhancing treatment-naïve glioma. This included IDH-mutated gliomas – 5 CNS WHO grade 4, 5 CNS WHO grade 3, and 4 CNS WHO grade 2 - consisting of 36% oligodendrogliomas and 64% astrocytomas, which were compared to 19 IDH wild-type glioblastomas (CNS WHO grade 4). A detailed overview of the molecular stratification is given in Fig. 2; Table 1.

### Metabolic and Spatial tumour characteristics

**Fig. 3:**
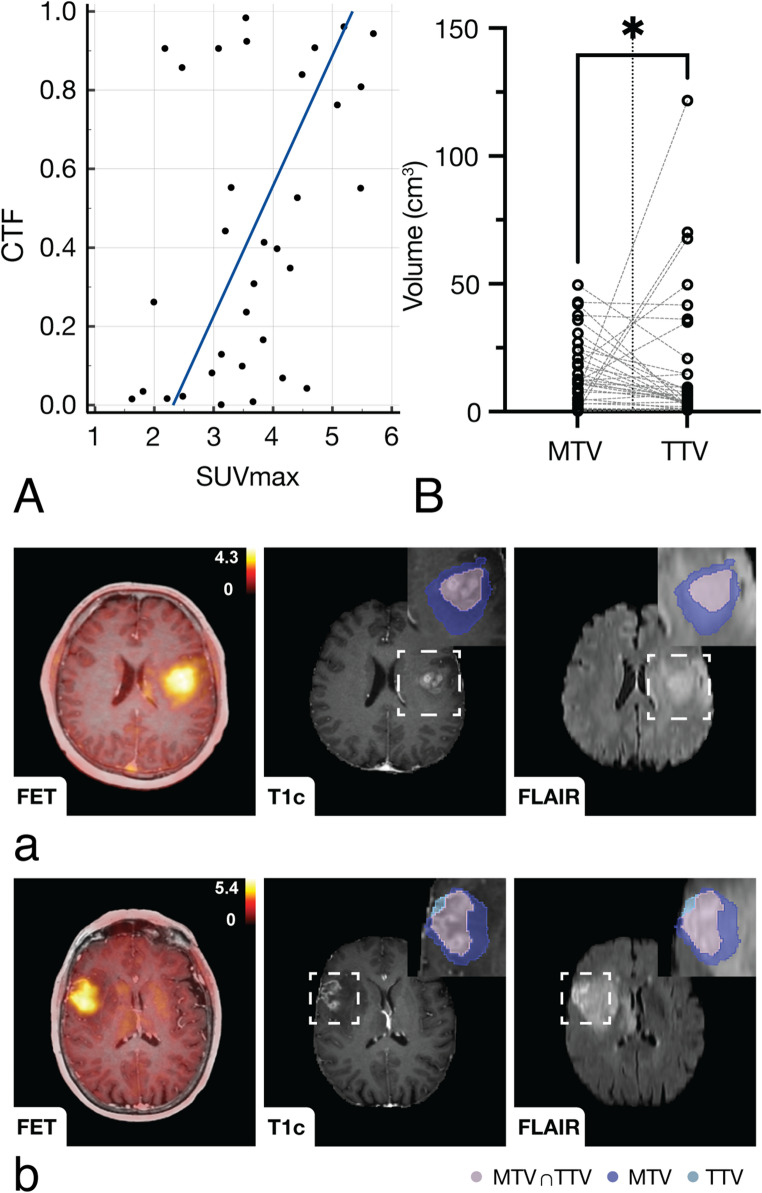
Volumetric analysis of spatial tumour characteristics and amino acid metabolism. **A** Contrast-enhancing tumour fraction (CTF) from structural MRI shows a significant association (*p* <.05) with amino acid uptake ([18 F]FET-PET) in glioma. **B** Metabolic tumour volume (MTV) surpassed total tumour volume (TTV) (*p* =.033, *n* = 33). (**a)** and (**b**) show representative examples of glioblastoma, IDH wild-type (CNS WHO grade 4), showing metabolically-active tumour that extends beyond the structurally apparent tumour margins. *p*-value < .05 was considered statistically significant

**Fig. 4:**
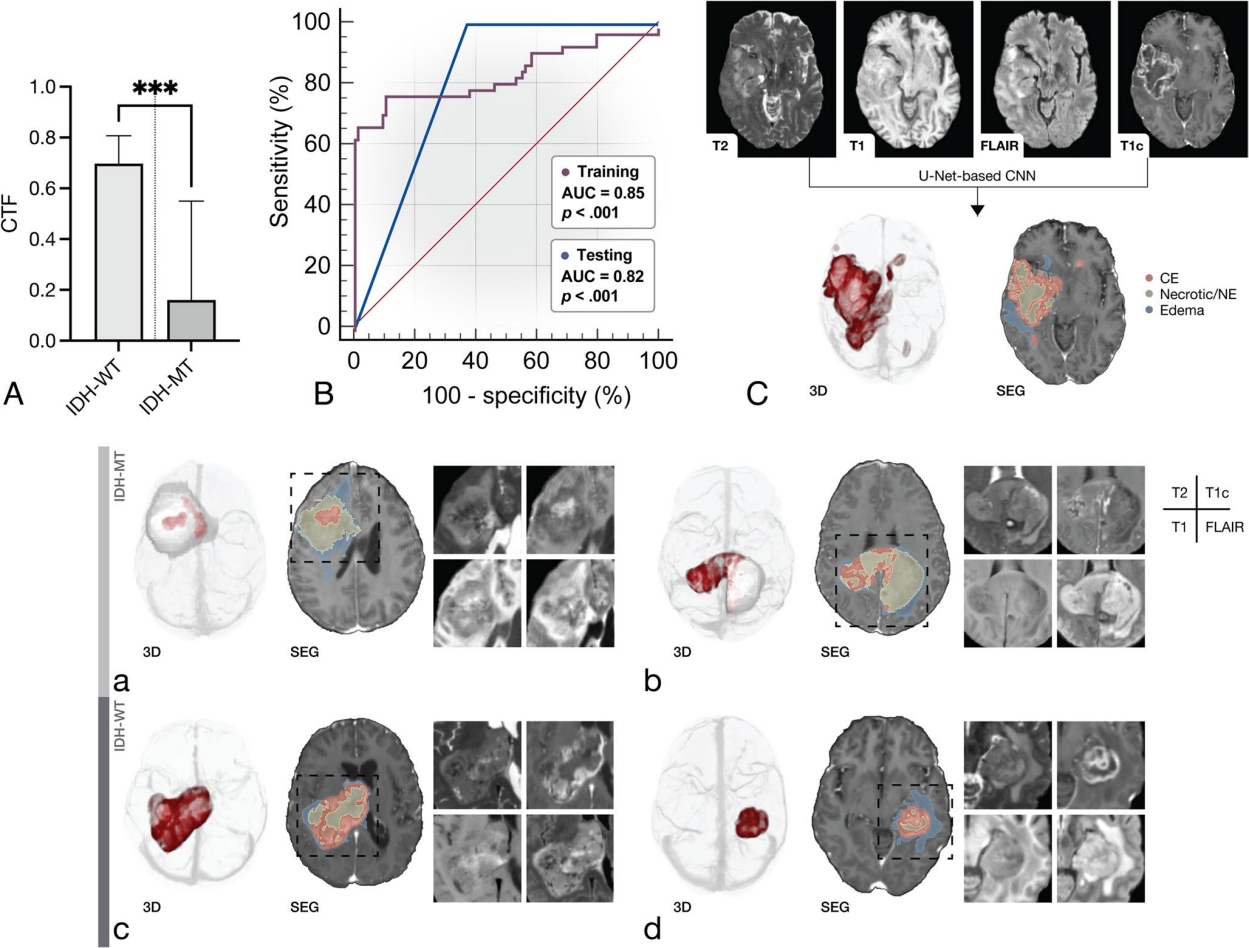
Evaluation of contrast-enhancing tumour fraction (CTF) for predictive IDH-genotyping. **A** Compared to IDH wild-type glioblastoma, IDH-mutated gliomas exhibited significantly lower CTF values (adjusted *p* <.001). **B** Classification of the IDH mutational status using the CTF presents excellent diagnostic performance (AUC ± SE = 0.85 ± 0.04, *p* <.001) based on Receiver Operating Characteristic analysis with similar results in the validation dataset. **C** Illustration of automated morphometry using an U-Net-based CNN based on a clinical standard MRI protocol. Illustration of exemplary cases of high-grade astrocytoma IDH-mutant (CNS WHO grade 4) (**a**-**b**), which exhibit a low CTF, as the non-CE mass is characteristically greater than the CE tumour (*p* <.001) in IDH-mutant glioma. Glioblastoma, IDH-wild-type (CNS WHO grade 4) (**c-d**) demonstrate an inverse relationship, consecutively yielding a higher CTF. IDH-MT/-WT = Isocitrate dehydrogenase mutated/wild-type, CE = Contrast-enhancing, NE = Non-enhancing. *p*-value < .05 was considered statistically significant

Measures of metabolic activity from [18 F]FET-PET, including SUVmean (Spearman rank correlation coefficient, 0.40; *p* =.022) and SUVmax (Spearman rank correlation coefficient, 0.42; *p* =.015), showed a significant correlation with the CTF (see Fig. 3). The CTF demonstrated lower values in IDH-mutated gliomas (median, 0.16 [Q1–Q3, 0.05–0.55; *n* = 49] vs. median, 0.70 [Q1–Q3, 0.61–0.81; *n* = 98], adjusted *p* <.001, *U*, 728) compared to IDH wild-type glioblastoma, as shown in Fig. 4 (A). This reflects the larger non-CE mass present in IDH-mutated gliomas (median, 21.10 cm3 [Q1–Q3, 3.44–56.07]) relative to the CE mass (median, 2.66 cm3 [Q1–Q3, 0.84–100.20], adjusted *p* <.001, *n* = 49, W, −811), as shown in Fig. 4, whereas IDH wild-type glioblastoma were characterised by an inverse relationship with greater CE tumour (median, 20.50 cm3 [Q1–Q3, 9.95–33.20] vs. median, 8.10 cm3 [Q1–Q3, 3.02–19.35], adjusted *p* <.001, *n* = 98, *W*, 4291). The structurally apparent TTV (median, 6.12 cm3 [Q1–Q3, 2.61–17.75]) was exceeded by MTV (median, 12.14 cm3 [Q1–Q3, 4.80-24.13]; *p* =.033, *n* = 33, W, 207), with a moderate Dice coefficient overlap (median, 0.40 [Q1–Q3, 0.21–0.54]). Considering that oligodendroglioma and astrocytoma are distinct molecular entities, we additionally investigated differences in CTF in the TCGA-LGG cohort [22] (Supplemental Fig. 1) using the same methodology as described above. As shown in Supplemental Fig. 2, IDH-mutated oligodendroglioma present lower CTF compared to astrocytoma (median, 0.03 [Q1–Q3, 0.01–0.27, *n* = 32] vs. median, 0.11 [Q1–Q3, 0.02–0.66, *n* = 24], *p* =.035, *U*, 274).

### Prediction of the IDH-genotype

When the CTF was evaluated for predictive IDH-genotyping, excellent diagnostic performance (AUC ± SE = 0.85 ± 0.04, *p* <.001, 95%-CI = 0.78–0.90; IDH wild-type, 98; IDH-mutated, 49) was apparent in the model building dataset (see Fig. 4, B), which determined an optimal threshold of ≤ 0.55 (sensitivity, 78%, specificity, 90%, accuracy = 86%, +LR = 7.6, –LR = 0.25) based on the Youden index. The corresponding confusion matrix yielded a PPV = 79% and NPV = 89%, based on a condition prevalence of 33%. Similar results were obtained using the original unmodified segmentations (AUC ± SE = 0.85 ± 0.04, *p* <.001), as shown in Supplemental Fig. 3. Next, the diagnostic test performance was validated in the PET/MRI cohort (AUC ± SE = 0.82 ± 0.06, *p* <.001, 95%-CI = 0.64–0.93, IDH wild-type, 19; IDH-mutated, 14), confirming the robustness as a predictive marker in an independent dataset. When the thresholds for CE (*p* >.05) and non-CE mass (AUC ± SE = 0.75 ± 0.07, *p* <.001) from the model-building dataset were evaluated in the validation cohort, the combined CTF demonstrated greater predictive power than its individual components (*p* <.001). Since IDH-mutated tumours are more commonly found in younger adults, and metabolic imaging has proven useful for predicting IDH status, we further evaluated the combined use of the CTF with SUVmax and patient age (< 49 years, based on the Youden index). Incorporating patient age (ΔAUC = 0.12, *p* =.02) or SUVmax (ΔAUC = 0.09, *p* >.05) further improved the classification compared to CTF alone. A subgroup analysis within CNS WHO grade 4 tumours showed that CTF discriminated IDH-mutant glioma from IDH-wild-type glioblastoma with AUC ± SE = 0.82 ± 0.06 (*p* <.001) at the pre-specified threshold of ≤ 0.55 (sensitivity, 74%, specificity, 90%, accuracy = 90%, +LR = 7.3, –LR = 0.29) based on the Youden index. The corresponding confusion matrix yielded a PPV = 67% and NPV = 93%, based on a subgroup condition prevalence of 22%. Comparable performance was observed in the validation cohort (AUC ± SE = 0.82 ± 0.06, *p* <.001). In the validation cohort, a follow-up analysis limited to either IDH-mutant oligodendroglioma (AUC ± SE = 0.82 ± 0.06, *p* <.001, +LR = 7.6, –LR = 0.25) or IDH-mutant astrocytoma (AUC ± SE = 0.82 ± 0.06, *p* <.001), respectively compared against IDH wild-type glioblastoma (CNS WHO grade 4), suggested no difference in diagnostic test performance.

There was no difference in CTF between three independent groups of randomly selected glioblastoma IDH wild-type samples (*p* >.05; Kruskal–Wallis test, 1.384), as shown in Supplemental Fig. 4. An overview of diagnostic measures is available in Table 2.

**Table 2:** Diagnostic measures from receiver operating characteristic (ROC) analysis

			AUC ± SE(*P*-value)	Threshold	Sensitivity	Specificity
Training cohort	IDH-MT vs. IDH-WT	CTF	0.85 ± 0.04(*p* <.001)	≤ 0.55	78%	90%
		CE mass	0.84 ± 0.04(*p* <.001)	< 6.43 mm^3^	71%	89%
		non-CE mass	0.64 ± 0.06(*p* =.009)	> 30.08 cm^3^	45%	91%
		Age	0.87 ± 0.03(*p* <.001)	≤ 49 y	78%	85%
	Glioblastoma vs. IDH-mutated glioma of CNS WHO grade 4	CTF	0.82 ± 0.06(*p* <.001)	≤ 0.55	74%	90%
Testing (validation) cohort	IDH-MT vs. IDH-WT	CTF	0.82 ± 0.06(*p* <.001)	≤ 0.55	100%	63%
		CE mass	0.50 ± 0.00(*p* >.05)	< 6.43 cm^3^	–	–
		non-CE mass	0.75 ± 0.07(*p* <.001)	> 30.08 cm^3^	50%	100%
		SUVmax	0.71 ± 0.09(*p* =.02)	≤ 3.85	86%	53%
		CTF + Age	0.93 ± 0.04(*p* <.001)	CTF ≤ 0.55, Age ≤ 49 y	79%	95%
		CTF + SUV	0.91 ± 0.05(*p* <.001)	CTF ≤ 0.55, SUVmax	86%	84%
	Glioblastoma vs. Oligodendroglioma	CTF	0.82 ± 0.06(*p* <.001)	≤ 0.55	–	–
	Glioblastoma vs. Astrocytoma	CTF	0.82 ± 0.06(*p* <.001)	≤ 0.55	–	–
	Glioblastoma vs. IDH-mutated glioma of CNS WHO grade 4	CTF	0.82 ± 0.06(*p* <.001)	≤ 0.55	–	–

*AUC* Area Under the Curve, *SE* Standard Error, *IDH-MT/-WT* Isocitrate dehydrogenase mutated/wild-type, *CNS* Central Nervous System, *WHO* World Health Organization, *CTF* Contrast-enhancing tumour fraction, *CE* Contrast-enhancing, *SUV* Standardised Uptake Value. *p*-value < .05 was considered statistically significant.

## Discussion

We performed automated morphometry of treatment-naïve glioma using an U-Net-based CNN and introduced the CTF for the assessment of spatial tumour characteristics in treatment-naïve glioma. First, we showed that metabolic activity from [18 F]FET-PET is significantly correlated with the CTF, linking spatial and metabolic tumour characteristics. We then demonstrated that IDH-mutated gliomas have a markedly lower CTF due to a predominance of non-contrast-enhancing tumour masses, in contrast to IDH wild-type glioblastoma. The CTF demonstrated excellent diagnostic accuracy for predictive IDH-genotyping in an independent model building and validation dataset, while outperforming individual CE and non-CE volumes. Combining the CTF with patient age as a clinical predictor or SUVmax from [18 F]FET PET further improved the predictive performance. Diagnostic accuracy was consistent across IDH-mutated subtypes and in a subgroup analysis of CNS WHO grade 4 tumours, demonstrating that the CTF enables molecular discrimination that extends beyond simply differentiating high-grade from low-grade tumours. MTV identified from [18 F]FET PET exceeded the structurally apparent tumour volume, highlighting the higher sensitivity of metabolic imaging in detecting diffuse infiltration, and thus, total tumour mass. In summary, the CTF is a novel non-invasive biomarker that captures spatial tumour characteristics and is closely associated with tumour metabolic activity, enabling robust preoperative IDH prediction. Its performance is enhanced when combined with quantitative [18 F]FET PET or clinical predictors, facilitating the personalised management of diffuse gliomas.

Metabolic dysfunction in glioma has a profound impact on its structural characteristics [29]. Key hallmarks of glioma, such as necrosis, arise as a consequence of severe hypoxia, which develops when tumour growth outpaces vascular supply and compensatory mechanisms become insufficient. In response to hypoxia, hypoxia-inducible factor 1α (HIF1α) is up-regulated, initiating a cascade that promotes metabolic adaptation and the expression of pro-angiogenic factors, most notably vascular endothelial growth factor (VEGF) [30]. This HIF1α-VEGF-driven pathway stimulates compensatory angiogenesis, resulting in the formation of aberrant, leaky vasculature that may manifest as contrast enhancement or increased perfusion on MRI [31]. The degree of metabolic dysregulation correlates closely with tumour aggressiveness: slow-growing gliomas, which typically harbour IDH mutations, often exhibit a larger proportion of non-enhancing tumour tissue, reflecting relatively preserved metabolic homeostasis and vascular integrity with lower levels of hypoxia. Notably, in IDH-mutant gliomas, the accumulation of the oncometabolite (R)−2-hydroxyglutarate ((R)−2HG) enhances the activity of EGLN prolyl 4-hydroxylases [32], leading to increased degradation of HIF1α; this reduction in HIF1α levels is thought to suppress angiogenesis and may partially help to explain the less aggressive clinical course observed in some of these tumours.

In contrast, highly proliferative neoplasms such as IDH wild-type glioblastoma display more pronounced metabolic dysfunction, characterised by extensive hypoxia, necrosis, and aberrant vascularisation, consequently presenting larger contrast-enhancing tumour volume. These macrostructural differences, fundamentally driven by underlying metabolic alterations, underscore the potential of imaging-based biomarkers to non-invasively predict molecular and metabolic phenotypes in glioma [14, 15, 18, 33].

Van Lent et al. (2020) showed that individual structural MRI signs lack sufficient accuracy to predict IDH status [34]. One notable exception is the T2-FLAIR mismatch sign, which has shown high specificity but limited sensitivity (42%) for identifying IDH-mutated, 1p/19q-intact low-grade gliomas [35].

More recently, Negro et al. (2024) reported that a structured, multi-parameter VASARI 2.0 lexicon may achieve high accuracy for IDH prediction in a retrospective, single-centre cohort [36], suggesting that rigorously standardised and combined visual features may be informative on IDH mutational status. However, the study cohort was imbalanced (22 IDH-mutant vs. 104 wild-type cases), and the skew was only mitigated by synthetic oversampling of the training split with ROSE (Random Over-Sampling Examples); moreover, the study lacked external or prospective validation – both factors limit the generalisability of these findings. Notably, the “tumour-enhancing proportion” (VASARI feature 5) was identified as a predictor of IDH genotype. Although conceptually similar to CTF, the VASARI “tumour-enhancing proportion” feature is derived from manual 2-D ROI estimates and categorical ranges, whereas CTF uses automated volumetric segmentation, yielding a more accurate, objective and reproducible metric – which may account for its higher diagnostic accuracy.

While advanced imaging techniques, such as 2-hydroxyglutarate magnetic resonance spectroscopy [37, 38], perfusion MRI [39], diffusion MRI [40], radiomics [41–43], or CEST [44] were demonstrated to show correspondence to the IDH-genotype, the requirement of either time-demanding measurements or complex data analysis have hindered clinical translation. On the other hand, the current study’s approach promotes clinical translation by using clinical standard MRI protocols and a publicly available U-Net-based CNN. As opposed to other deep learning techniques, such as machine learning or radiomics analysis, which are often perceived as a “black box” by clinicians, the herein proposed biomarker is biologically meaningful and interpretable.

Due to the retrospective nature of the study, conclusions should be interpreted with caution until validated by prospective studies. We acknowledge the potential limitations introduced by differing IDH testing methods. Future studies may benefit from uniform sequencing-based assessment, though the current approach is aligned with contemporary clinical practice and WHO diagnostic standards, this reflects real-world diagnostic variability and reinforces the robustness of our model in clinically relevant settings. Because the findings of this study are restricted to contrast-enhancing tumours, advanced imaging methods, such as MRS, diffusion-weighted or perfusion MRI, with additional [18 F]FET PET, may offer additional insights into the IDH genotype in suspected non-enhancing gliomas. Furthermore, the current study’s approach is unsuitable for longitudinal imaging of therapy response to IDH-targeted therapy. The finding that [18 F]FET PET–derived metabolic tumour volume (MTV) exceeds the structurally apparent tumour volume aligns with previous reports highlighting the superior sensitivity of metabolic imaging in detecting infiltrative tumour components [45–47]. Future research should leverage this strength by employing multimodal training approaches that integrate structural MRI and metabolic PET data as ground truth. Deep learning models trained on combined datasets may improve the prediction of tumour margins and the approximation of TTV from MRI alone, potentially enabling more accurate, PET-informed segmentation in centres without routine access to amino acid PET. Furthermore, future investigations should be dedicated towards multimodal and -parametric approaches for IDH prediction.

## Conclusion

We performed spatial phenotyping of treatment-naïve contrast-enhancing glioma using an U-Net based CNN and proposed a non-invasive biomarker for the assessment of spatial tumour characteristics. We evaluated and validated its diagnostic potential for predictive IDH-genotyping in an independent cohort where it enabled robust IDH-classification with excellent diagnostic accuracy – with implications for the clinical management of adult-type glioma.

## Supplementary Information

Below is the link to the electronic supplementary material.

ESM 1 (PDF 132 KB)

ESM 2 (DOCX 1.42 MB)

## Data Availability

The datasets generated during and/or analysed during the current study are available from the corresponding author on reasonable request. The dataset from The University of California San Francisco Preoperative Diffuse Glioma MRI (UCSF-PDGM) cohort is publicly available. DeepBraTumIA (https://www.nitrc.org/projects/deepbratumia*)* is publicly available.

## Abbreviations

IDH-MT/-WT: isocitrate dehydrogenase mutated/wild-type
MGMT: O6-methylguanine-DNA methyltransferase
LOH1p/19q: loss of heterozygosity on chromosomes 1p and 19q
HGG/LGG: high-/low-grade glioma
