# Supplementary material for: Spatial tumour characteristics as an indirect marker of metabolic dysregulation: evaluation for non-invasive IDH-genotyping of glioma using hybrid [18 F]FET-PET/MRI
Source: Eur J Nucl Med Mol Imaging. 2025 Aug 28;53(3):1939–50. doi: 10.1007/s00259-025-07520-8 (PMC12860868; doi:10.1007/s00259-025-07520-8)
Supplement: Supplementary file 1 — (PDF 132 KB) [file 259_2025_7520_MOESM1_ESM.pdf]

STARD 2015 Checklist

Section & Topic	No.	Item	✔ (N/A)
**TITLE OR ABSTRACT**			
	1	Identification as a study of diagnostic accuracy using at least one measure of accuracy (such as sensitivity, specificity, predictive values, or AUC)	X
**ABSTRACT**			
	2	Structured summary of study design, methods, results, and conclusions (for specific guidance, see STARD for Abstracts)	X
**INTRODUCTION**			
	3	Scientific & clinical background, including the intended use and clinical role of the index test	X
	4	Study objectives and hypotheses	X
**METHODS**			
Study design	5	Whether data collection was planned before the index test and reference standard were performed (prospective study) or after (retrospective study)	X
Participants	6	Eligibility criteria	X
	7	On what basis potentially eligible participants were identified (such as symptoms, results from previous tests, inclusion in registry)	X
	8	Where and when potentially eligible participants were identified (setting, location and dates)	X
	9	Whether participants formed a consecutive, random or convenience series	X
Test methods	10a	Index test, in sufficient detail to allow replication	X
	10b	Reference standard, in sufficient detail to allow replication	X
	11	Rationale for choosing the reference standard (if alternatives exist)	N/A
	12a	Definition of and rationale for test positivity cut-offs or result categories of the index test, distinguishing pre-specified from exploratory	X
	12b	Definition of and rationale for test positivity cut-offs or result categories of the reference standard, distinguishing pre-specified from exploratory	X
	13a	Whether clinical information and reference standard results were available to the performers/readers of the index test	X
	13b	Whether clinical information and index test results were available to the assessors of the reference standard	X
Analysis	14	Methods for estimating or comparing measures of diagnostic accuracy	X
	15	How indeterminate index test or reference standard results were handled	N/A
	16	How missing data on the index test and reference standard were handled	N/A
	17	Any analyses of variability in diagnostic accuracy, distinguishing pre-specified from exploratory	N/A
	18	Intended sample size and how it was determined	N/A
**RESULTS**			
Participants	19	Flow of participants, using a diagram. Include the figure number (preferably figure 1) or page number	X
	20	Baseline demographic and clinical characteristics of participants	X
	21a	Distribution of severity of disease in those with the target condition	X
	21b	Distribution of alternative diagnoses in those without the target condition	N/A
	22	Time interval and any clinical interventions between index test and reference standard	N/A
Test results	23	Cross tabulation of the index test results (or their distribution) by the results of the reference standard	N/A
	24	Estimates of diagnostic accuracy and their precision (such as 95% confidence intervals)	X
	25	Any adverse events from performing the index test or the reference standard	N/A
**DISCUSSION**			
	26	Study limitations, including sources of potential bias, statistical uncertainty, and generalisability	X
	27	Implications for practice, including the intended use and clinical role of the index test	X
**OTHER INFORMATION**			
	28	Registration number and name of registry	N/A
	29	Where the full study protocol can be accessed	N/A
	30	Sources of funding and other support; role of funders	X

*N/A stands for not applicable and may be a reasonable choice depending on the type of study performed
