# Supplementary material for: Spatial tumour characteristics as an indirect marker of metabolic dysregulation: evaluation for non-invasive IDH-genotyping of glioma using hybrid [18 F]FET-PET/MRI
Source: Eur J Nucl Med Mol Imaging. 2025 Aug 28;53(3):1939–50. doi: 10.1007/s00259-025-07520-8 (PMC12860868; doi:10.1007/s00259-025-07520-8)
Supplement: Supplementary file 2 — (DOCX 1.42 MB) [file 259_2025_7520_MOESM2_ESM.docx]

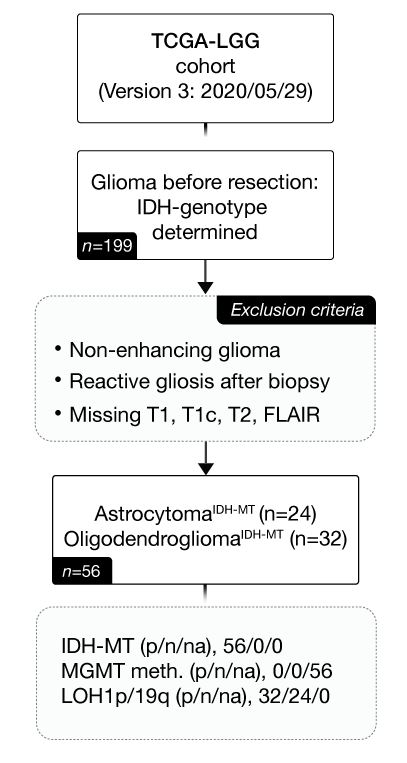


Figure 1. **Selection criteria in the** **TCGA-LGG cohort.** Fifty-six subjects with confirmed and contrast-enhancing IDH-mutated astrocytoma and oligodendroglioma prior to resection were included based on the eligibility criteria. Patients with mixed glioma and those with non-standard MRI protocols (missing T1, T1c, T2, or FLAIR sequences) were excluded. IDH-MT/-WT = Isocitrate dehydrogenase mutated/wild-type, MGMT = O6-methylguanine-DNA-methyltransferase, LOH1p/19q = Loss of heterozygosity of 1p/19q. p/n/na = positive/negative/not applicable.


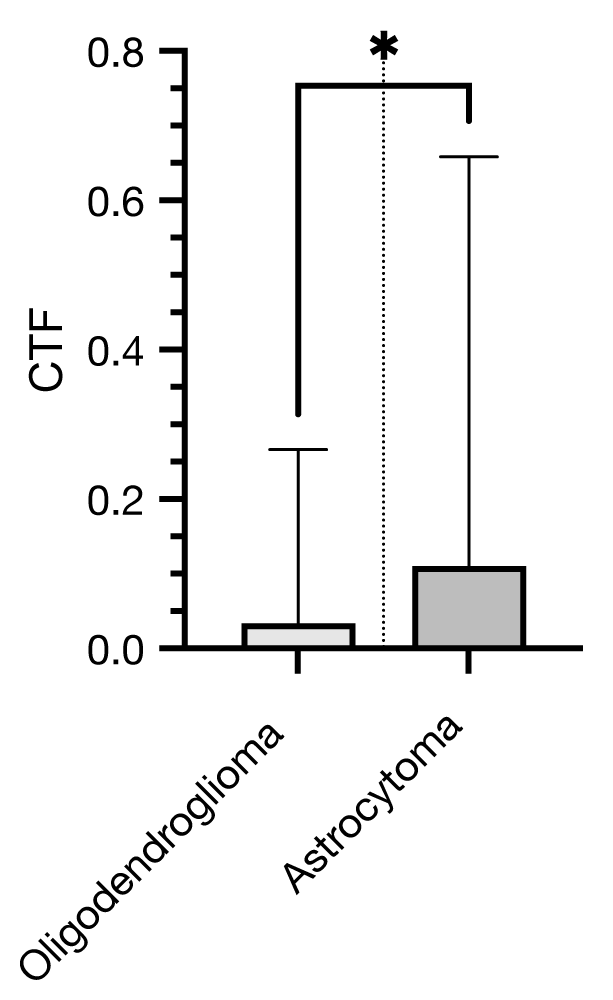


Figure 2. **Comparative evaluation of** **contrast-enhancing tumour fraction (CTF) between oligodendroglioma and astrocytoma.** IDH-mutated oligodendroglioma present lower CTF compared to astrocytoma (median, 0.03 [IQR, 0.26, n = 32] vs. median, 0.11 [IQR 0.64, n = 24], *p* = .035, U, 274). *p*-value <  .05 was considered statistically significant.


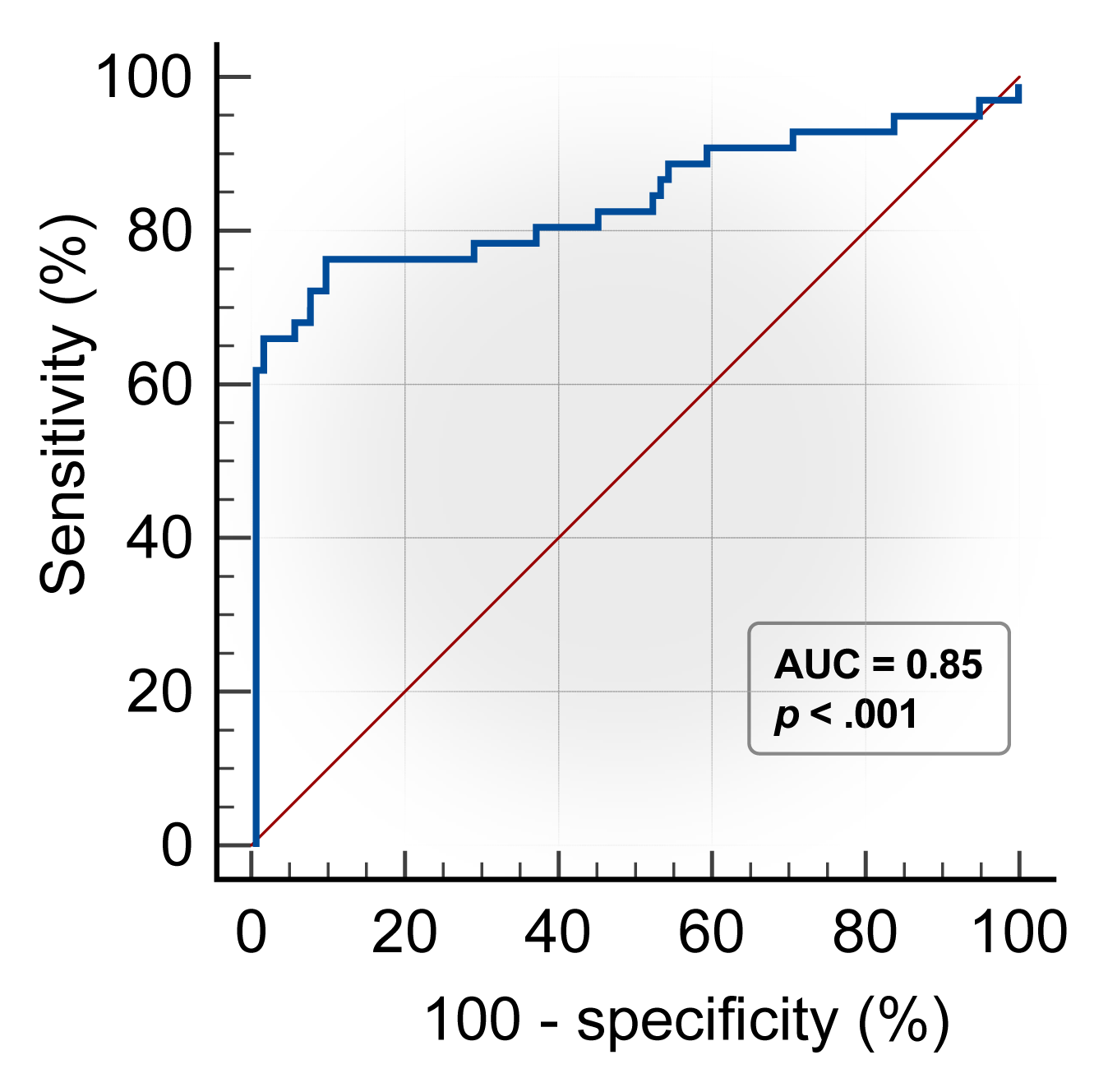


Figure 3. **ROC analysis** **using unmodified automated morphometry**. Classification of IDH mutational status using contrast-enhancing tumour fraction (CTF) shows comparable diagnostic performance (AUC ± SE = 0.85 ± 0.04, *p* < .001, 95%-CI = 0.78-0.90; IDH wild-type, 98; IDH-mutated, 48) to manually corrected segmentations with a threshold at ≤0,55. The detection of (subtle) contrast-enhancement failed in one subject. AUC = Area Under the Curve. *p*-value <  .05 was considered statistically significant.


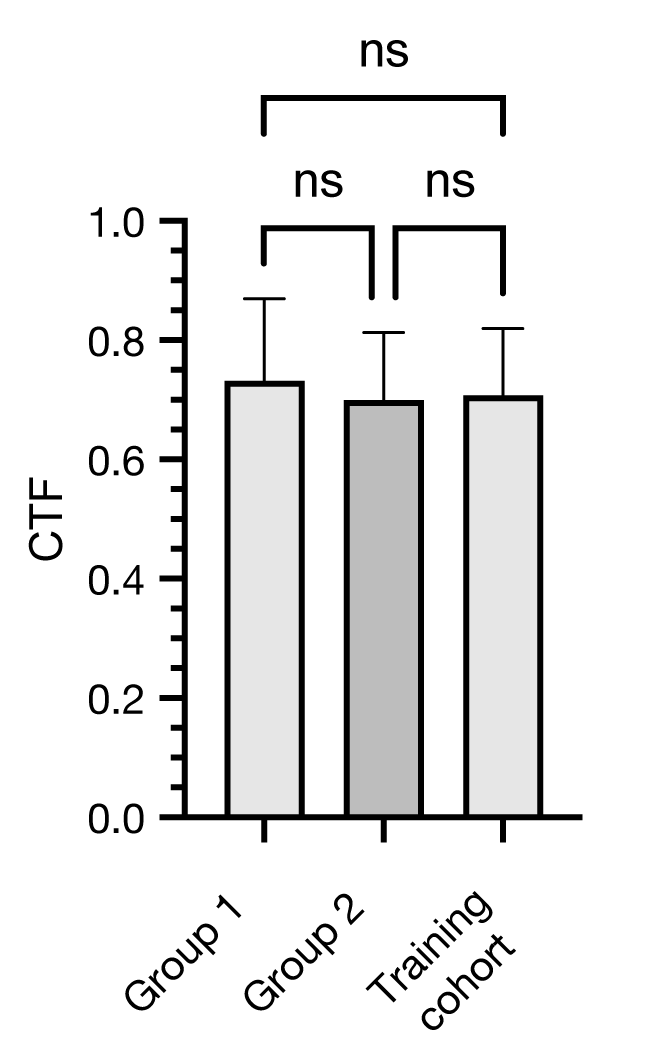


Figure 4. **Comparative analysis of contrast-enhancing tumour fraction (CTF) with unmodified results for different glioblastoma IDH wild-type groups from the** **UCSF-PDGM cohort.** After exclusion of the IDH wild-type samples used in the discovery cohort (n=98), two additional groups of randomly selected glioblastoma (respectively n=98) were analysed. The detection of non-CE mass failed in one subject. There was no difference in CTF between three independent groups of randomly selected glioblastoma samples (*p* > .05; Kruskal-Wallis test, 1.384). *p*-value <  .05 was considered statistically significant.
